# Analyzing Medical Research Results Based on Synthetic Data and Their Relation to Real Data Results: Systematic Comparison From Five Observational Studies

**DOI:** 10.2196/16492

**Published:** 2020-02-20

**Authors:** Anat Reiner Benaim, Ronit Almog, Yuri Gorelik, Irit Hochberg, Laila Nassar, Tanya Mashiach, Mogher Khamaisi, Yael Lurie, Zaher S Azzam, Johad Khoury, Daniel Kurnik, Rafael Beyar

**Affiliations:** 1 Clinical Epidemiology Unit Rambam Health Care Campus Haifa Israel; 2 School of Public Health University of Haifa Haifa Israel; 3 Department of Internal Medicine D Rambam Health Care Campus Haifa Israel; 4 Institute of Endocrinology, Diabetes and Metabolism Rambam Health Care Campus Haifa Israel; 5 The Ruth & Bruce Rappaport Faculty of Medicine Technion-Israel Institute of Technology Haifa Israel; 6 Clinical Pharmacology and Toxicology Section Rambam Health Care Campus Haifa Israel; 7 Diabetes Stem Cell Laboratory Rambam Health Care Campus Haifa Israel; 8 Department of Internal Medicine B Rambam Health Care Campus Haifa Israel; 9 The Ruth & Bruce Rappaport Faculty of Medicine and Rappaport Research Institute Technion-Israel Institute of Technology Haifa Israel; 10 Clinical Pharmacology Unit Rambam Health Care Campus Haifa Israel; 11 Rambam Health Care Campus Haifa Israel

**Keywords:** synthetic data, electronic medical records, MDClone, validation study, big data analysis

## Abstract

**Background:**

Privacy restrictions limit access to protected patient-derived health information for research purposes. Consequently, data anonymization is required to allow researchers data access for initial analysis before granting institutional review board approval. A system installed and activated at our institution enables synthetic data generation that mimics data from real electronic medical records, wherein only fictitious patients are listed.

**Objective:**

This paper aimed to validate the results obtained when analyzing synthetic structured data for medical research. A comprehensive validation process concerning meaningful clinical questions and various types of data was conducted to assess the accuracy and precision of statistical estimates derived from synthetic patient data.

**Methods:**

A cross-hospital project was conducted to validate results obtained from synthetic data produced for five contemporary studies on various topics. For each study, results derived from synthetic data were compared with those based on real data. In addition, repeatedly generated synthetic datasets were used to estimate the bias and stability of results obtained from synthetic data.

**Results:**

This study demonstrated that results derived from synthetic data were predictive of results from real data. When the number of patients was large relative to the number of variables used, highly accurate and strongly consistent results were observed between synthetic and real data. For studies based on smaller populations that accounted for confounders and modifiers by multivariate models, predictions were of moderate accuracy, yet clear trends were correctly observed.

**Conclusions:**

The use of synthetic structured data provides a close estimate to real data results and is thus a powerful tool in shaping research hypotheses and accessing estimated analyses, without risking patient privacy. Synthetic data enable broad access to data (eg, for out-of-organization researchers), and rapid, safe, and repeatable analysis of data in hospitals or other health organizations where patient privacy is a primary value.

## Introduction

### Background

Access to large databases of electronic medical records (EMRs) for research purposes is limited by privacy restriction, security laws and regulations, and organizational guidelines imposed because of the assumed value of the data. It, therefore, requires approval of the local institutional review board (IRB), but this regulatory process is often time consuming, thereby delaying research and imposing difficulties on data sharing and collaborations. In addition, researchers could apply for a research grant if preliminary data could be extracted and analyzed before making an IRB application, but this is impossible if the data are inaccessible.

Consequently, data anonymization, namely, making reidentification of patients impossible, is required to balance the risk of privacy intrusions with research accessibility. Establishing effective anonymization techniques will promote the future release of data for global access, envisioning democratization of data for all researchers, and facilitate the use of real-world data as a base for study.

One approach for preventing identification of personal records is data masking, namely, removal of identifying information from a dataset, so that individual data cannot be linked with specific individuals. Other techniques include pseudoanonymization, in which a coded reference is attached to a record instead of identifying information, and aggregation, in which data are displayed as totals [1,2]. However, nonaggregation techniques still pose the risk of exposing individuals, as shown by multiple reports [3-7]. In addition to preventing any initial exploration of the data before the IRB approval, once the approval is granted, the researcher may still be blocked by regulatory and ethical barriers for sharing, transferring, and securing the stored data. An alternative approach is the generation of realistic synthetic records comprising the same statistical characteristics and time-dependent properties as the original data such that their analysis yields the same results without mapping the data elements to actual individuals. Thus, synthetic data that are prepared properly can achieve full and irreversible anonymization.

However, creating synthetic data that not only ensure privacy but also retain the information needed for analysis is far from trivial. Kartoun [8,9] proposes a methodology for generating virtual patient repositories, termed electronic medical records bots (EMRBots), based on configurations of population-level and patient-level characteristics. He explains that although such repositories are of high value for training and education, developing computational methods, and assisting hackathons, they cannot serve for studying and predicting real patient outcomes, as their creation does not account for combinations of associations and time-dependent interactions. To reliably mimic EMRs, linear and nonlinear relationships between the variables as well as the temporal arrangement of medical events must be considered.

Other systems for generating synthetic data assume that the data are selected from common distributions that do not comply with the characteristics of real-world medical data and, therefore, may not retain the correlations between multiple variables. Furthermore, they may use prior knowledge of the anticipated relationships, thereby limiting the possibility of true discovery [10-15]. The Synthea system (MITRE Corporation, Massachusetts) [3,14] models care processes and outcomes for several clinical conditions along with their progression. It relies on publicly available datasets and health statistics and synthesizes data according to clinical guidelines and expertise, thereby potentially reflecting ideal scenarios that are not sufficient to replace real EMRs. The Observational Medical Dataset Simulator (OSIM) [15] offers to synthesize data related to diseases and drugs, based on probability distributions estimated from real data, while accounting for time, gender, and age. Relationships are restricted by OSIM to behave in a specific format as reflected by the estimated transitional probability matrix, thereby limiting the ability to reflect other and more complex relationships.

Recently, autoencoders, a technique based on unsupervised deep learning models, has been proposed for synthesizing patient data. By assuming a large enough patient population, autoencoders can learn a representation of the data and then generate a representation that is close to the original input. For instance, medGAN [16] uses real patient records as input to generate high-dimensional discrete samples through a combination of autoencoders and generative adversarial networks. Although this method shows promise in terms of imitating distributional measures and predictions [16,17], it can synthesize only count and binary variables and ignores the longitudinal nature of medical events. Furthermore, a limited privacy risk was observed, and thus, autoencoders cannot yet be considered safe.

This paper studied the validity of synthetic data generated by the MDClone system (Beer-Sheba, Israel), which synthesizes data based directly on the actual real data of interest. The real data is automatically queried from the EMR data lake just before the synthesis. The system was implemented in a number of studies at our institution, Rambam Health Care Campus, located in Haifa, Israel. Our institution is a 1000-bed tertiary academic hospital in Northern Israel and has been using a proprietary EMR system since 2000 (Prometheus, developed by the hospital’s department of information technology). Validating the use of synthetic data for research necessitates a comparison of the results derived from synthetic data with those based on the original data. Previous validation studies on synthetic health data are scarce, of limited scope, and are typically concerned with secondary uses of the data that have minor clinical implications [3,11,14,18,19]. Furthermore, little has been done to compare the statistical results of synthetic data with those of real data [3,14,19]. A more comprehensive validation process concerning meaningful clinical questions and various types of data and outcomes is required for establishing the suitability of synthetic health data for medical research.

We conducted a cross-hospital study to validate the results obtained from synthetic data in various clinical research projects. This paper presents the validation results for five studies conducted at our institution, concerning omission of recommended medication, effect of time to procedure and of hospitalization measures on postdischarge survival, imaging-related risks, and comparison of diabetic treatments. IRB approval to use real data was received, allowing comparative analysis of real vs synthetic data. These studies were used to assess the accuracy and precision of statistical estimates derived from synthetic patient data. The studies represented various population sizes, types of variables and statistical modeling and were based on the hospital’s EMR records routinely generated from 2007 to 2017.

### The Synthetic Data Generating System

The MDClone system was used in this study for generating synthetic data. This system has been installed in our institute’s information technology platform since 2017, and its implementation includes the generation of a structured data lake, a query tool, and a synthetic data generator. The data lake integrates the EMR records with all hospital data sources relating to patient visits, hospitalizations, coded diagnoses, medications, surgical and other procedures, laboratory tests, demographics, and administrative information. The data are presented in an anonymous and standardized format (Health Insurance Portability and Accountability Act of 1996 style). The query engine allows the retrieval of a wide range of variables, in a defined time frame, around an index event. Once an IRB authorization has been granted, the system enables the eligible investigators seamless access to real data structure and analysis with respect to the authorized dataset [20-22]. Otherwise, the system provides the investigator with easy access to synthetic data by the defined query, without revealing the real patient data.

The algorithm used for generating synthetic data is multivariate in nature and generates all variables together, using a covariance measure. It maintains multivariate relationships even on subpopulations of the data (see demonstration in Multimedia Appendices 1-3), as long as they are not too small to expose individual subjects. It does so without assuming any specific form of the underlying distributions and can accept any input, allowing for the discovery of relationships not known before loading the data.

The algorithm treats categorical variables at the first step, ensuring the use of values not unique to a small number of patients. If a subpopulation is identified as unique, such that patients could be identified by certain variables, the values of these variables are censored from the data for these patients. The algorithm then proceeds to extract statistical characteristics from the data, which are used to generate synthetic data with similar properties.

The generation of synthetic data is performed by random sampling from statistical distributions estimated from the original data; thus, each round of data synthesis based on the same query yields a different cohort with similar statistical features. To verify the reliability and validity of the synthetic data, the system produces a report with (1) censoring rate for each variable; (2) a summary of the distribution of each variable, original vs synthetic; and (3) a comparison of all pairwise correlations.

## Methods

### Validation Methodology

For each participating study, we repeatedly produced five synthetic datasets based on the query to be used to extract the real data. We then statistically analyzed each set and compared the results, namely, the effect point estimates and their uncertainty levels, as reflected by the confidence intervals, with those obtained from the real data. The types of effects compared included proportions, odds ratios, hazard ratios, and survival curves, as obtained by applying the relevant statistical models.

In addition, to evaluate the stability of results obtained from synthetic data, we evaluated the consistency of the estimates across the synthetic sets. Although an initial impression was obtained from observing the results across the five synthetic sets, we repeatedly generated numerous synthetic sets to evaluate the bias and stability of the estimates. To obtain small enough standard errors, 1000 repetitions were used. Bias was defined by the difference between the mean across all synthetic sets and the estimate obtained from the real data. Stability was evaluated by the range of this difference. The bias and stability were evaluated for three of the studies, which represented the types of statistical outcomes addressed in this study, and reflected the common measures used in clinical research: proportions (the Proton Pump Inhibitors [PPIs] Prescription Study), hazard ratios and survival curves (the Percutaneous Coronary Intervention [PCI] and ST-Elevation Myocardial Infarction [STEMI] Study), and odds ratios (the Hypoglycemia Insulin Study).

### Generation of Synthetic Data

For each participating study, the following steps were taken throughout the analysis:

- The investigator logged into the system and defined the patient cohort by setting inclusion and exclusion criteria.
- The information required for these patients was defined by a query. An approximation for the number of patients meeting the criteria was then provided by the system. The researcher could define a reference event (eg, the first myocardial infarction event) that could be used to pull data in relative temporal terms (eg, the last hospitalization before the event). Any data included in the hospital’s EMR could be requested, provided it was within the access definitions for the researcher, as set by an administrator.
- The cohort with its defined data was extracted and seamlessly converted into synthetic information with the same structure as the original data. A data file was prepared and downloaded, along with a report providing a descriptive comparison between the synthetic data and the original data for each variable.
- The synthetic data were statistically analyzed.
- Following IRB approval, real data were extracted and analyzed using the same analytics.

### Participating Studies

A total of five clinical studies conducted in the hospital were selected for the validation process. The studies addressed contemporary topics with important clinical and medical implications. They represented a range of statistical questions, types of analysis, and population sizes that are frequently confronted in hospital research. Tables describing the real populations are provided in Multimedia Appendices 4-7, and synthetic data files are provided in Multimedia Appendices 8 and 9.

#### The Proportion of Omission of Proton Pump Inhibitor Prescriptions for Gastroprotection

Gastrointestinal bleeding is one of the most common preventable adverse drug events [23,24], and antiplatelet and anticoagulant medications are the most common drugs associated with hospitalization caused by PPI prescription errors [25]. To reduce the risk of gastrointestinal bleeding, guidelines recommend prescribing PPIs to high-risk patients [26,27]. This study assessed the proportion of PPI omission for gastroprotection in patients discharged with prescribed combinations of oral anticoagulants (OACs; warfarin, dabigatran, rivaroxaban, or apixaban) and antiplatelets (aspirin, clopidogrel, prasugrel, or ticagrelor), accounting for additional indications for prophylactic PPI use (age >65 years and concomitant steroid use). In each subgroup, we examined the proportion of patients with recommended administration of concomitant PPIs.

#### The Effect of Time to Percutaneous Coronary Intervention in ST-Elevation Myocardial Infarction Patients on Death and Heart Failure

This study examined the effect of door-to-balloon time (D2B) among STEMI patients on the occurrence of congestive heart failure (CHF) or mortality, within 180 days of catheterization. According to the guidelines adopted in Israel in 2014, PCI should be performed within 90 min of arrival to the hospital [28]. Kaplan-Meier survival rate estimates were calculated, and the effect of D2B and STEMI-associated factors [29,30] was estimated by a multivariate Cox proportional hazard regression, accounting for other adverse events, such as severe cardiac presentation (cardiogenic shock, cardiac arrest, ventricular fibrillation, ventricular tachycardia, and complete atrioventricular block) and prior ischemic heart disease (IHD; previous coronary artery bypass surgery, myocardial infarction, and PCI). In addition, laboratory test results indicating low hemoglobin (≤10), high creatinine (>1), and high blood urea nitrogen (BUN; >30), as well as potential confounders (age, gender, and year), were all accounted for.

#### The Impact of Blood Urea Nitrogen on Postdischarge Mortality Among Patients With Acute Decompensated Heart Failure

Acute decompensated heart failure (ADHF) is the leading cause of hospital admission in patients older than 65 years [31]. This study investigated the effect of BUN levels during hospitalization on 3-year mortality after discharge from the hospital among patients with ADHF. Admission and discharge BUN levels were extracted. The predictive value of BUN for mortality was evaluated using multivariate Cox proportional hazard regression, accounting for the number of associated comorbidities. In addition, the levels of brain natriuretic peptide, red cell distribution width, and blood sodium were included in the model as dichotomous variables, in accordance with accepted thresholds.

#### The Risk of Nephropathy Following Magnetic Resonance Imaging Using Gadolinium-Based Contrast Agents Compared With the Risk Following Computed Tomography–Based Imaging Using Iodine-Based Contrast Agents

Contrast-induced nephropathy (CIN) following iodine-based contrast-enhanced imaging has been widely known as a leading cause of acute kidney injury (AKI) [32-34]. This study aimed to establish the risk of AKI following contrast-enhanced magnetic resonance imaging (MRI) relative to that of contrast-induced computed tomography (CT). We included all adult patients who had undergone a contrast-enhanced CT or MRI. Propensity score matching was used to account for known risk factors for CIN and AKI by applying nearest-neighbor 1:4 matching between MRI and CT patients. Comorbidities such as diabetes and IHD and the target organ of the imaging study were also accounted for. AKI rates were compared by odds ratios calculated from the full data and the matched data by the Fisher exact test and the Mantel-Haenszel test, respectively.

#### The Risk of Hypoglycemia in Patients With Diabetes Treated by Detemir or Glargine Insulins by Blood Albumin Level

Detemir and glargine are long-acting insulins commonly used for inpatient treatment [35]. However, detemir is albumin bound, raising a concern for increased risk of hypoglycemia for patients with hypoalbuminemia [36,37], and guidelines for treating hyperglycemia do not prefer one insulin over the other [35]. This study assessed the risk of hypoglycemia in patients with low albumin treated with insulin detemir vs glargine. Retrieved data included all adult patients treated with detemir or glargine and laboratory results for albumin, creatinine, and glucose levels during a 5-day time frame. In addition, age, gender, weight, insulin dose, insulin dose-to-weight ratio, home usage of insulin, receiving of short insulin, division of hospital stay, and length of stay were also accounted for. Hypoglycemia risks were estimated by fitting a multivariate logistic regression model that included main effects and second-order interactions as the predictors and hypoglycemia (glucose level <70 mg/dL) as the dependent variable. Variables were selected for the model by a stepwise procedure based on the Akaike Information Criterion.

## Results

### Protein Pump Inhibitors Prescription Study

Between 2007 and 2017, we identified 12,188 patients discharged on OACs, some of whom additionally received a single antiplatelet, either aspirin (n=3953) or P2Y ADP receptor blockers (clopidogrel, prasugrel, or ticagrelor) antiplatelet therapy (n=882), or a double antiplatelet therapy (DAT; n=417).

**Figure 1:**
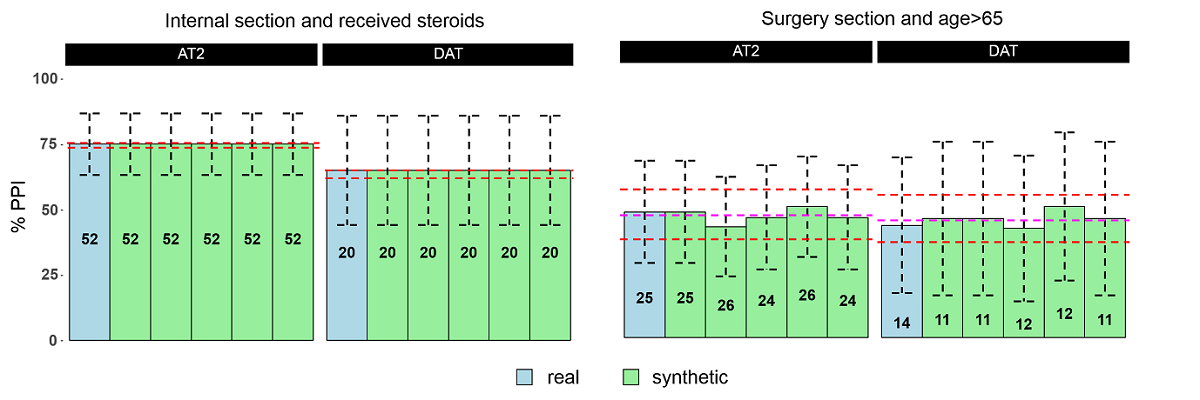
PPI administration (%) for patients receiving the clopidogrel, prasugrel or ticagrelor antiplatelet (AT2) or dual antiplatelet (DAT). The total number of patients in the subgroups are given inside the bars. If no censoring was required (left panel – Internal Section patients that received steroids), proportions of PPI administration calculated from the synthetic sets were essentially identical to the proportions in the real data, and their range across 1000 sets (minimum and maximum in red dotted lines) was very narrow. If censoring was required, as in the case of the Surgery Section, results varied across the synthetic sets, and their ranges were wider (right panel – Surgery Section patients older than 65 years). The means across 1000 sets (purple lines) show small biases.

Comparisons between results obtained from five synthetic sets and the real data are shown in Figure 1. Overall, good predictions of the real data results were obtained from the synthetic data. For most subgroups, such as patients discharged from internal medicine departments with OAC and antiplatelets and receiving concomitant steroids, generating synthetic data did not require censoring, as no observations were found to be unique. Estimates from synthetic data were, therefore, identical to those from real data, regardless of sample size, including their uncertainty levels, as reflected by the confidence intervals (left panel). On the basis of repeated runs of 1000 synthetic sets, the PPI administration proportions for this subgroup were highly stable, as indicated by the nearly zero bias and their very narrow range across the 1000 repeats.

For small subgroups, some instability was observed, as can be readily seen by the estimates obtained from the five synthetic sets (right panel). The estimates’ range across the 1000 synthetic sets was wider for those two subgroups (minimum −10.5% and maximum +8.5%). Their overall mean across 1000 sets shows biases of −1.3% and 1.9% for AT2 and DAT, respectively, which are small when compared with the uncertainty level (reflected by the confidence intervals) of the estimates from real data.

### Percutaneous Coronary Intervention and ST-Elevation Myocardial Infarction Study

Between 2013 and 2016, 597 patients diagnosed with STEMI who underwent primary PCI were identified, excluding cases in which more than 6 hours had passed before performing primary PCI or with CHF before intervention. Boolean classifications were used to extract information on patient conditions: the variable *severe cardiac presentation* indicated cardiogenic shock, cardiac arrest, ventricular fibrillation, ventricular tachycardia, or atrioventricular block on admission, and the variable *prior ischemic heart disease* indicated prior coronary artery bypass surgery, myocardial infarction, or PCI.

**Figure 2:**
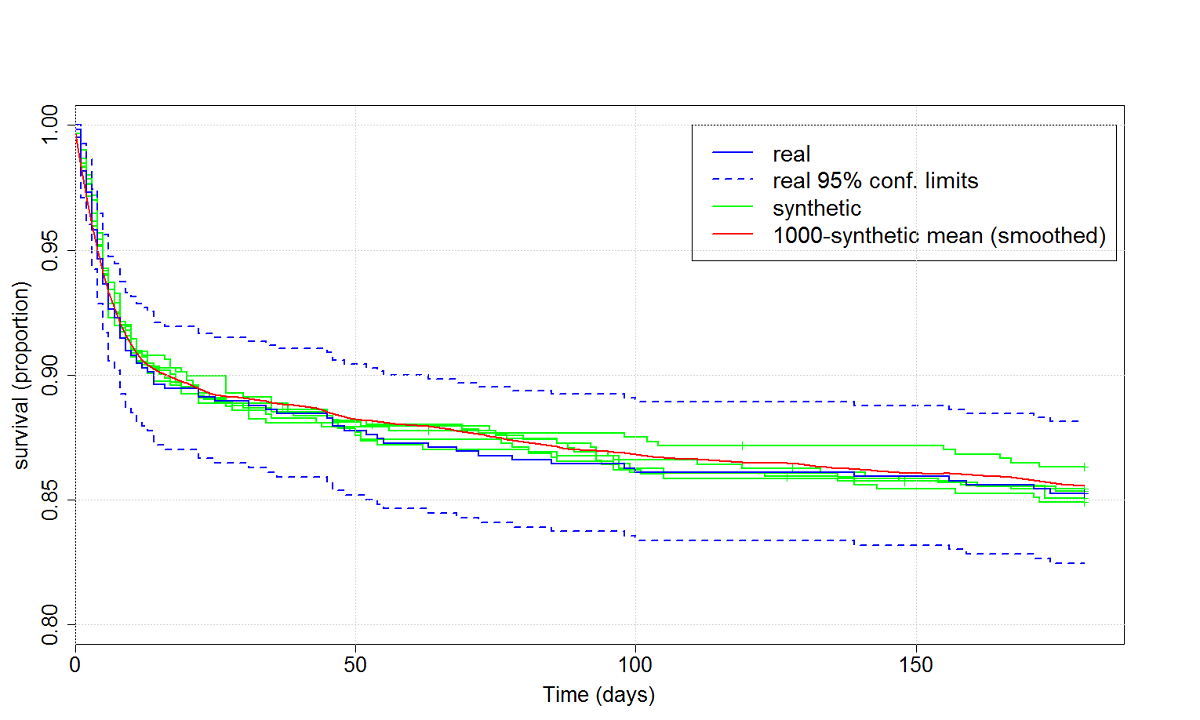
Kaplan-Meier 180-day event-free (CHF/mortality) survival curves after primary PCI, estimated from the real data with 95% confidence limits (blue) and from five repeatedly generated synthetic datasets (green). Survival curves based on synthetic data were similar to curves based on real data, and the mean curve based on 1000 synthetic sets was similar to the curve obtained from the real data.

**Figure 3:**
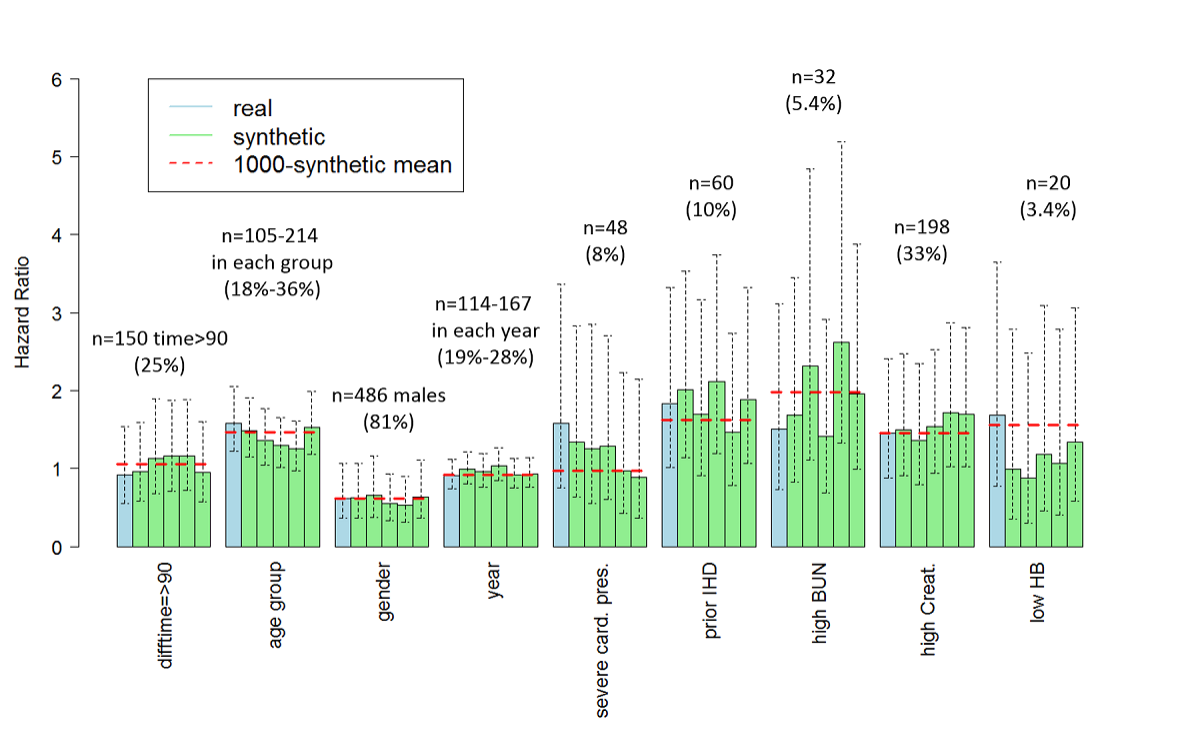
Hazard ratios with 95% confidence intervals for CHF or mortality within 180 days of primary PCI based on real data (blue) and on five synthetic datasets (green). For each variable, the number of cases and percentage in the real data is given. Conclusions were typically consistent between the real and the synthetic data, and across the synthetic sets. In the case of increased risk with age, some variability was observed. The mean result across 1000 synthetic sets (dotted red line) for results with high confidence, was close to the result from the real data, implying small bias.

Survival curves estimated from synthetic data were similar to the curves estimated from real data with little variability between curves obtained from the five synthetic sets (Figure 2) and were within the confidence limits obtained from the real data. The mean curve based on 1000 synthetic sets was similar to the curve obtained from the real data. Hazard ratios for 180 event-free (CHF/death) days are shown in Figure 3. A D2B greater than 90 min revealed no increased risk, based on either the real or the synthetic data. Conclusions were typically consistent between real and synthetic data and across the five synthetic sets. Estimates were also consistent in the uncertainty level (width of confidence intervals). In the case of increased risk with age and borderline significance for a slight increase in risk for patients with prior IHD, as obtained from the real data, some variability was observed. For results with higher confidence, the hazard ratio estimates were more stable. Yet, the bias of the estimate obtained from synthetic data, as estimated by 1000 repeatedly generated synthetic sets, was small when compared with the uncertainty of the estimate from real data. As expected, the stability and the bias of the synthetic results were better for variables with narrower confidence intervals (age group, gender, and year) compared with variables with wider confidence intervals (prior IHD and high BUN).

Importantly, all estimates obtained from synthetic data, for the survival curve and the hazard ratios, were within the 95% confidence limits obtained from the real data, namely, within the range of potential values of the true survival rate and the true hazard ratio.

### Blood Urea Nitrogen and Acute Decompensated Heart Failure Study

**Figure 4:**
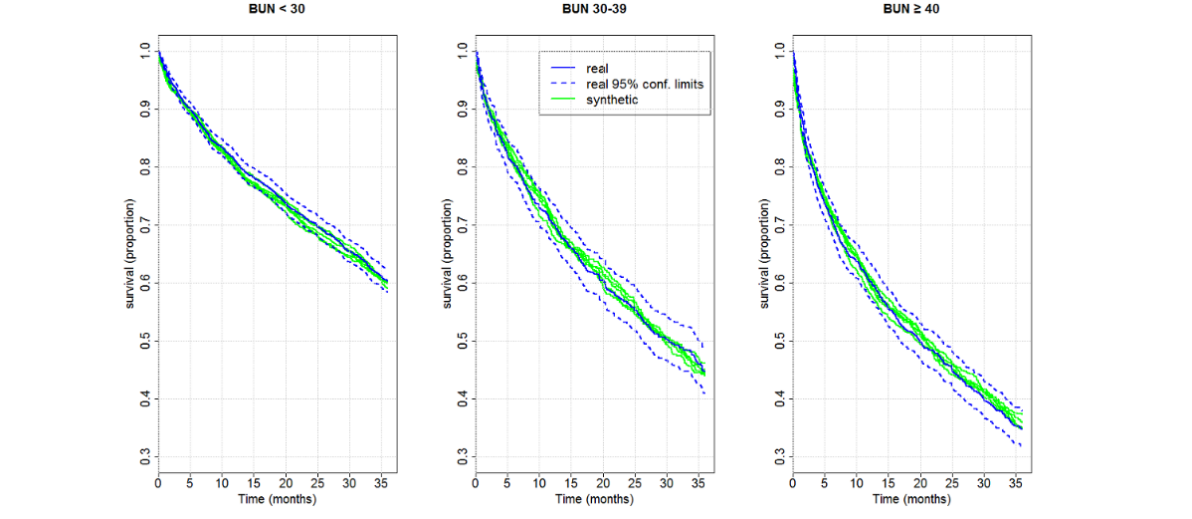
Kaplan-Meier three-year survival curves by admission BUN level, as estimated from the real data (in blue) and from five repeatedly generated synthetic datasets (in orange). The survival curves estimated from the synthetic sets were very close to the curve estimated from the real data.

**Figure 5:**
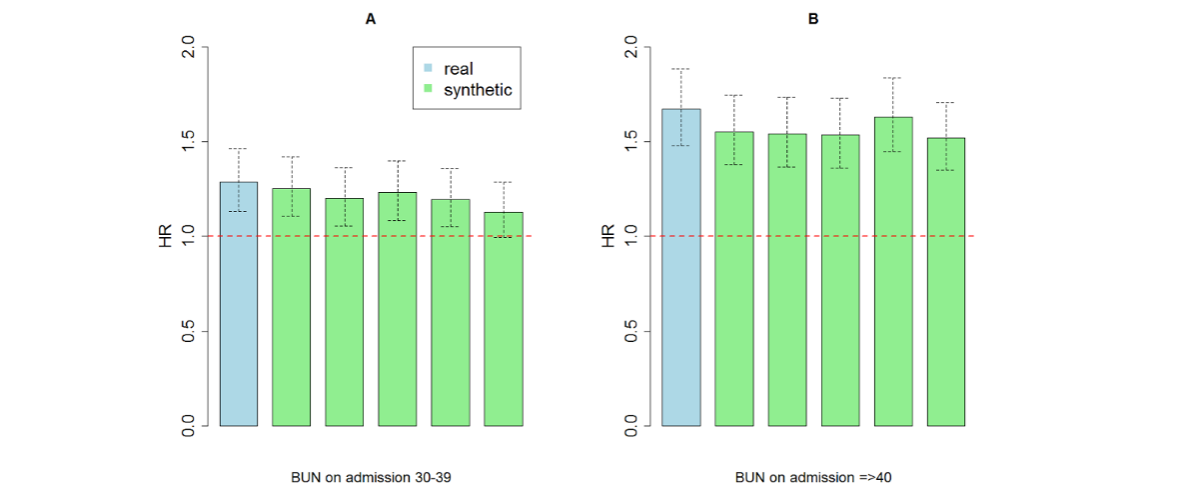
Hazard ratios with confidence intervals by admission BUN level, obtained by Cox proportional hazard regression based on real data and on five synthetic datasets. Hazard ratios relative to the reference group of BUN below 30 based on real data were 1.29 for patients with BUN between 30 and 39 (panel A) and 1.67 for patients with BUN 40 or above (panel B). Hazard ratios estimated from synthetic data were slightly lower. The width of confidence intervals was consistent between the real and the synthetic data, and across the synthetic sets.

Between 2007 and 2017, 4590 patients were hospitalized with a primary diagnosis of heart failure and survived to discharge. To limit the number of subgroups, a Boolean classification was used for extracting information on comorbidities instead of specific diagnoses. As shown in Figure 4, Kaplan-Meier 3-year survival obtained from the real data was nearly 60% for an admission BUN of below 30, 44% for BUN of 30 to 39, and 37% for BUN of 40 or above, implying that high admission BUN is a risk marker for mortality within 3 years. Similar estimates were obtained from the five synthetic sets. Hazard ratios relative to the *below 30* group were estimated from the real data as 1.29 for patients with BUN 30 to 39 and 1.67 for patients with BUN 40 or above. Hazard ratios estimated from synthetic data were slightly lower (Figure 5). As in the PCI-STEMI Study, all estimates from synthetic data, for the survival rate and the hazard ratios, were within the confidence limits obtained from the real data.

### Imaging Nephropathy Study

We identified 718 patients who underwent a contrast-enhanced MRI between 2013 and 2017 and 12,592 patients who underwent CT imaging between 2011 and 2017, excluding patients who underwent additional contrast-enhanced imaging within 3 days around the index imaging. To limit the number of subgroups, diagnoses and drugs were defined as Boolean variables.

**Figure 6:**
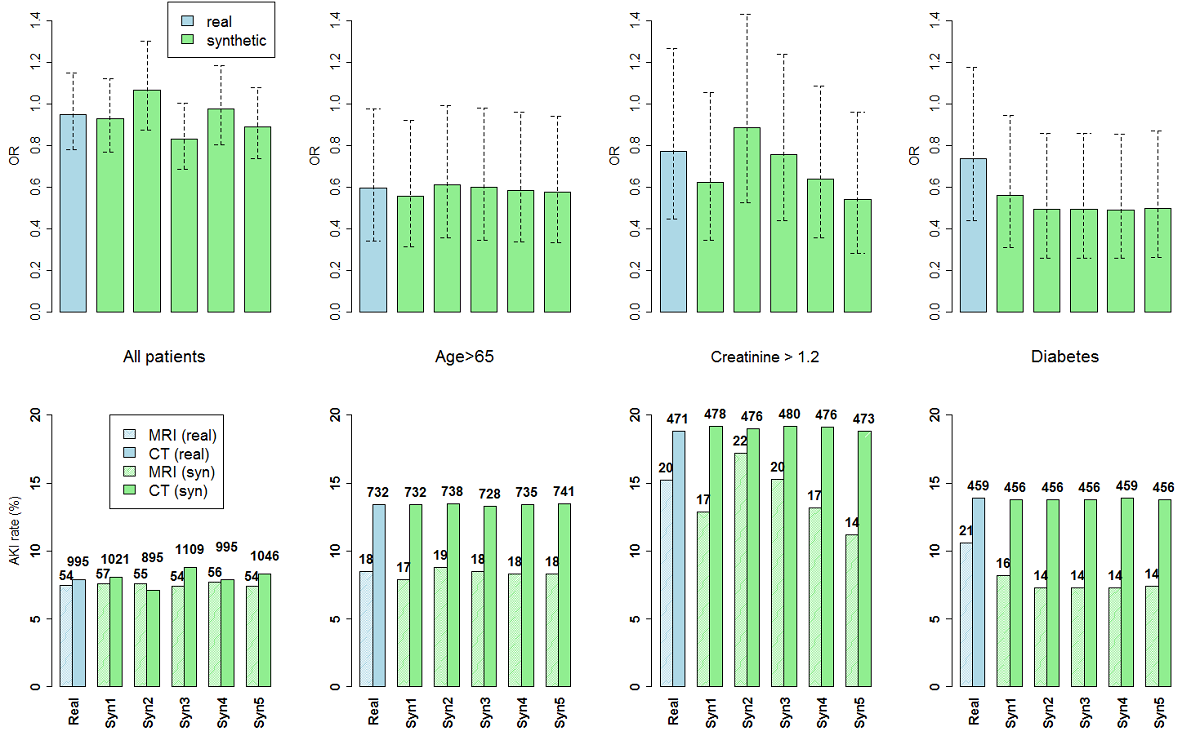
Acute kidney injury (AKI) rates (lower panel) and odds ratios with 95% confidence intervals (upper panel) in four different subgroups for the real data and five repeatedly generated synthetic datasets (Syn1-Syn5). The number of patients in the data for each subgroup is shown above the rate bars. Results obtained from the synthetic data were generally consistent with those obtained from the real data. AKI rates were well estimated for patients older than 65 years of age, and the borderline statistical difference remained consistent; AKI rate estimates were less stable for patients with high creatinine, yet the conclusion of no statistical difference was consistent; Odds ratios for diabetic patients were under-estimated due to under-estimated AKI rates for the very small number of diabetic patients that underwent MRI.

Odds ratios obtained from the real data and five synthetic sets are presented in Figure 6. For the relatively large CT group, AKI rates were consistent between the real and the synthetic sets and across the synthetic sets for all patient subgroups. For the small MRI group, the number of AKI cases per subgroup was only 18 to 21. The AKI rates were well estimated for patients older than 65 years, and the borderline statistical difference remained consistent; the AKI rate estimates were less stable for patients with high creatinine, yet the conclusion of no difference was consistent. For patients with diabetes, AKI rates and odds ratios were lower across all synthetic sets and should, therefore, be interpreted with caution. All odds ratio estimates obtained from synthetic data were within the 95% confidence limits obtained from the real data.

### Hypoglycemia Insulin Study

**Figure 7:**
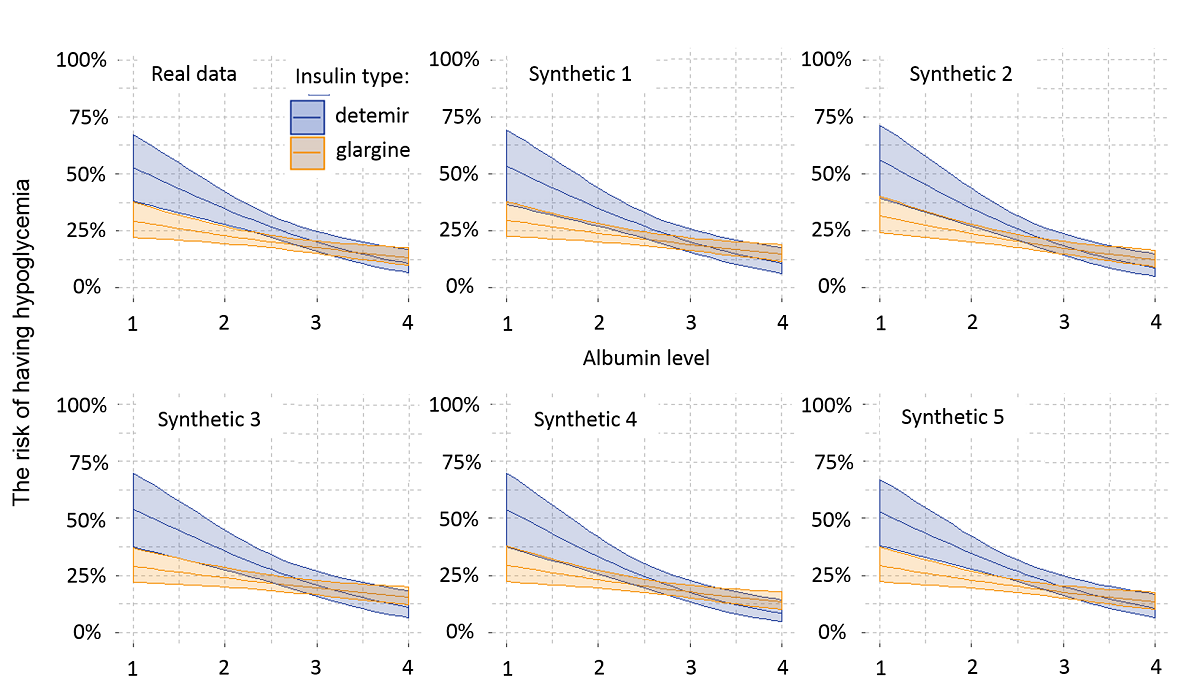
Risk predictions with 95% confidence intervals for detemir and glargine insulin treatments for a range of albumin values, based on the real data (top left) and five synthetic datasets (other panels). The risks estimated from the synthetic sets were highly similar to the curves estimated from the real data, and consistently indicated association of detemir use with a higher prevalence of hypoglycemic events in patients with hypoalbuminemia.

**Figure 8:**
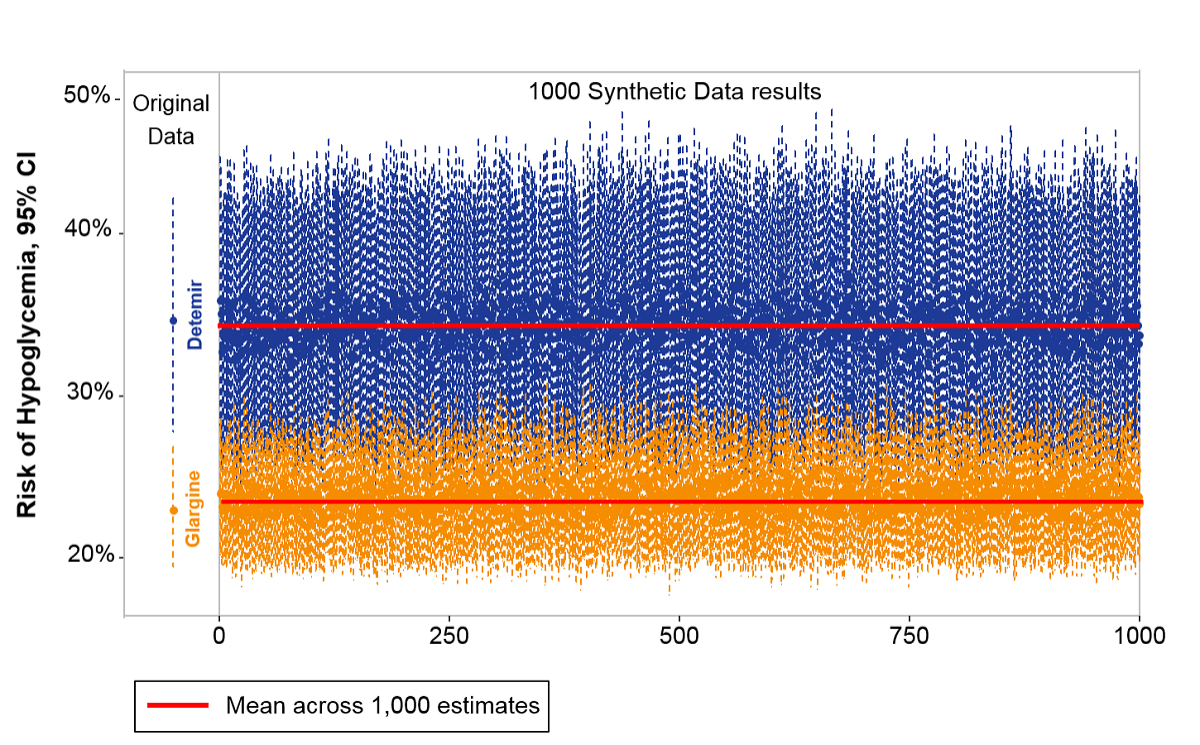
Risk predictions at albumin 2 gr/dL for 1000 repeatedly generated synthetic sets, compared to estimates obtained from the real sets (thin dotted line on the left marks the confidence intervals with the point estimates marked on the line). All synthetic sets predicted a higher hypoglycemia rate for detemir, and all were within the confidence limits of the estimates from the real data. The synthetic data estimates, as showed by their means (thick red lines), are biased from the real data estimates by –0.003 for detemir and by +0.006 for glargine.

Between 2012 and 2016, 4677 adult patients were hospitalized and treated with detemir (832/4677, 17.78%) or glargine (3844/4677, 82.19%) insulins. The risk curves estimated from the synthetic sets for detemir and glargine treatments across various albumin values (Figure 7) were highly similar to the curves estimated from the real data and consistently indicated the association of detemir use with a higher prevalence of hypoglycemic events in patients with hypoalbuminemia. Figure 8 presents risk predictions for 1000 repeatedly generated synthetic sets, compared with the estimates obtained from the real data. The estimates from all synthetic sets predicted a higher hypoglycemia rate for detemir and were within the confidence limits obtained from the real data. Their bias was −0.003 for detemir and +0.006 for glargine.

## Discussion

### Principal Findings

The use of synthetic data based on EMR is an approach for obtaining an estimate of real statistical results at a stage when real data are not available for the investigator. This paper examined the validity of statistical results based on synthetic data by comparison with real data for five studies, using medical records from our institution. Our study extended the scope of previous studies and investigated the performance of synthetic data under a variety of medical research questions. We used a system implemented in our institution that transforms the real data to synthetic data, which, when analyzed, provides the investigator with a reasonably accurate estimate of the real data results, and findings based on the synthetic data can be published in accordance with the institution policy. We assumed reliable performance of the system in privacy preservation, yet a future study aimed to investigate and validate issues related to the security and irreversibility of the synthetic data is of high relevance. Furthermore, sharing of synthetic data files that imitate particular real datasets and are generated within the hospital EMR platform must be a strategic decision of the hospital, accounting for concerns that are beyond academic considerations, such as costs of generating the data, timing of its release for sharing, and means of storage and access.

Five clinical studies on different topics, performed by separate research groups, were used for this validation study. The studies varied in population sizes and types of variables and statistical analysis. The validation study showed that the results derived from synthetic data were predictive of real data results. This was demonstrated with high consistency across all clinical studies. When the number of patients was large relative to the complexity and number of variables with very little or no censoring, as in the Hypoglycemia Insulin Study, the system proved itself highly predictive, with strong consistency of results between synthetic and real data, even for analyses involving complex computations and multiple stages such as stepwise logistic regression. Thus, the system can be effectively used to assess results from large data. Furthermore, when no censoring was imposed, precise predictions were obtained for proportions from synthetic data, regardless of sample size, as in the PPI Prescription Study.

For studies based on smaller populations that accounted for confounders and modifiers by multivariate models, such as the PCI-STEMI Study (n=597) and the Imaging Nephropathy Study (n=718), clear trends were still correctly observed by the synthetic data, although the predictions were of moderate accuracy. Nevertheless, these predictions are of high importance for guiding investigators before real data analysis and in generating a predictive hypothesis based on synthetic data that can then be applied to real data.

Several steps should be taken to minimize prediction bias caused by censoring when using synthetic data. Similar to any complex multivariate analysis, researchers should limit the number of variables to the minimum necessary and, when formulating the query, define variables to include information at the minimal required resolution, as in Boolean coding. When adhering to this recommendation, high consistency was achieved in the BUN-ADHF Study, which contained a large number of patients (n=4590) but also many subgroups. In addition, as seen in this study, analysis of multiple synthetic sets can guide the investigator by providing information on the stability of the synthetic results and indicating possible bias.

### Comparison With Prior Work

Previous validation studies on synthetic health data primarily considered secondary use of the data, with few medical implications [3,11,14,18,19]. Loong [19] did a limited comparison of statistical results between real and synthetic data, concluding that synthetic data are suitable for exploratory analysis. Walonoski et al [3] compared statistical properties with publicly available statistics for type 2 diabetes and found incorrect results for several variables, such as age at diagnosis, prevalence by racial groups, comorbidity rates, and survival, and acknowledged the need to increase the realistic level of the patient records. In a later paper, Chen et al [14] compared rates obtained from datasets generated by Synthea with publicly reported rates for four health care quality measures, showing inaccuracies that were partly caused by ignoring noncompliance with clinical guidelines and diversity in health care utilization.

Our validation study included a comprehensive validation process concerning meaningful clinical questions and various types of data and outcomes, which represent the scope of studies and type of statistical analysis conducted on hospital records. We used a system that seamlessly synthesizes data based on the actual original data of interest. We compared results obtained from the synthetic data with those obtained from the original data and included analysis of 1000 repeatedly generated synthetic datasets to estimate the bias and stability of the results.

### Limitations

Small populations may challenge the synthesis of data by (1) limiting the quality of the estimated statistical characteristics of the original data, particularly for high-dimensional multivariate distributions and outliers, and (2) causing selection bias in the estimates, if censoring of observations is made to prevent patient identification. Yet, as shown in this study, even varied and biased results obtained for very small subgroups, as in the Imaging Nephropathy Study, were still within the confidence limits of the results based on the original data. In addition, although interactions and correlations are preserved by the synthetic data, as shown in this study, high-order and complex relationships can be further investigated for very large study populations that involve hundreds or more variables, where the synthetic data results can also be compared with those generated by autoencoders.

Synthesis of nonstructured data, such as imaging results and free text from medical reports, has not yet been implemented in the synthesis engine and requires structuring of the data using image analysis, natural language processing, or other suitable approaches, enabling the eventual extraction of the statistical characteristics of the data. In addition, for some conditions considered in this paper, such as diabetes and CHF, structured data alone may be incomplete, and thus, extracting information from text can enhance the results on structured data.

Missing values for a particular variable in the original data are treated as a population subcategory by itself, for which statistical characteristics of all other variables are extracted separately. Thus, the synthesized data contain missing values for that subcategory as well. On obtaining the synthetic data, the researcher can decide if and how to impute the missing values, as in the case of real data.

### Conclusions

We provide a comprehensive evaluation of the use of synthetic data in comparison with real data, from an EMR data bank of a large academic medical center, based on five clinical studies conducted by five different research groups. In general, results based on synthetic data were highly predictive of those based on real data. Cases and conditions for which prediction may be nonprecise or biased were discussed and typically result from either censoring applied by the system to protect patient anonymity or data samples too small for quality estimation. Synthetic data, interpreted with an understanding of its limitations, are a powerful tool to guide clinical data analysis and research and allow for rapid, safe, and repeated analysis of routine data in a hospital setting and other health organizations where patient privacy is imperative.

## Appendix

Multimedia Appendix 1 Preservation of interactions and associations.

Multimedia Appendix 2 Spearman correlation coefficients for all pairs of numeric variables, based on the synthetic data (vertical axis) and the original data (horizontal axis). The correlation is preserved for the wide range of correlations, from negative to positive coefficients.

Multimedia Appendix 3 Boxplot of hemoglobin levels - comparison of MIMIC III (Original) and the synthetic datasets, by patient's age and hematocrit level. The high order correlation between hematocrit level, hemoglobin level and age, is consistent between the original data and the synthetic data. The delicate decline of hemoglobin as age increases, subject to the increase of hemoglobin level with hematocrit level, in general and within age group, is well preserved by the synthetic data.

Multimedia Appendix 4 Data Characteristics Table – PPI Prescription Study.

Multimedia Appendix 5 Data Characteristics Table – PCI-STEMI Study.

Multimedia Appendix 6 Data Characteristics Table – BUN-ADHF Study.

Multimedia Appendix 7 Data Characteristics Table – Hypoglycemia Insulin Study.

Multimedia Appendix 8 Synthetic data files and a variable description file - PPI Prescription study.

Multimedia Appendix 9 Synthetic data files and a variable description file - BUN-ADHF study.

## Abbreviations

ADHF: acute decompensated heart failure
AKI: acute kidney injury
BUN: blood urea nitrogen
CHF: congestive heart failure
CIN: contrast-induced nephropathy
CT: computed tomography
D2B: door-to-balloon time
DAT: double antiplatelet therapy
EMR: electronic medical record
IHD: ischemic heart disease
IRB: institutional review board
MRI: magnetic resonance imaging
OAC: oral anticoagulant
OSIM: Observational Medical Dataset Simulator
PCI: percutaneous coronary intervention
PPI: proton pump inhibitor
STEMI: ST-Elevation Myocardial Infarction
