# Supplementary material for: Analyzing Medical Research Results Based on Synthetic Data and Their Relation to Real Data Results: Systematic Comparison From Five Observational Studies
Source: JMIR Med Inform. 2020 Feb 20;8(2):e16492. doi: 10.2196/16492 (PMC7059086; doi:10.2196/16492)
Supplement: Multimedia Appendix 1 [file medinform_v8i2e16492_app1.docx]

## Supplement

# Medical research based on synthetic data is predictive of real data results: Systematic comparison from five observational studies

### Preservation of interactions and associations

A fundamental part of synthetic data generation is accounting for inter-variable interactions. By analyzing the original data and creating non-parametric models to represent the data, the distributional parameters are estimated. These models do not contain information that enables re-creation of the original data. Nevertheless, they are sufficient to generate a new, fictitious, population, retaining the original characteristics of the data, including the interactions between different variables. Non-numeric interaction are presented by replacing the discrete content of a variable with numbers, to enable the calculation of a rank correlation.

Temporal associations are preserved using referral time points, as follows. In each query, a reference event, such as a hospitalization or a surgery, is used, and other events are queried relative to the reference event, while preserving time gaps and time ratios. This ensures that time-related association are preserved. For instance, adverse effects or therapeutic effects that occur after the administration of a drug (reference event), maintain their temporal association with results of tests obtained before administering the drug.

As a demonstration for preserving the original correlation and interaction structure, we generated synthetic data for two original datasets. The first one includes various lab results such as systolic and diastolic blood pressure, hemoglobin, alkaline phosphatase, blood urea nitrogen (BUN), body temperature and white blood cells count, extracted from the hospital's EMR database for the same cohort used for the Imaging Nephropathy Study. Figure 1-S is a plot of all pairwise correlation coefficients calculated from the synthetic data, compared to the coefficients calculated from the real data. As is clearly visible, all points on the graph, corresponding to various magnitudes in both positive and negative directions, are either on the equality line or very close to it, indicating that the correlation is well preserved. This finding was statistically established by the signed rank test, testing the hypothesis that the mean difference in correlations was equal to 0 (p-value=0.7406).


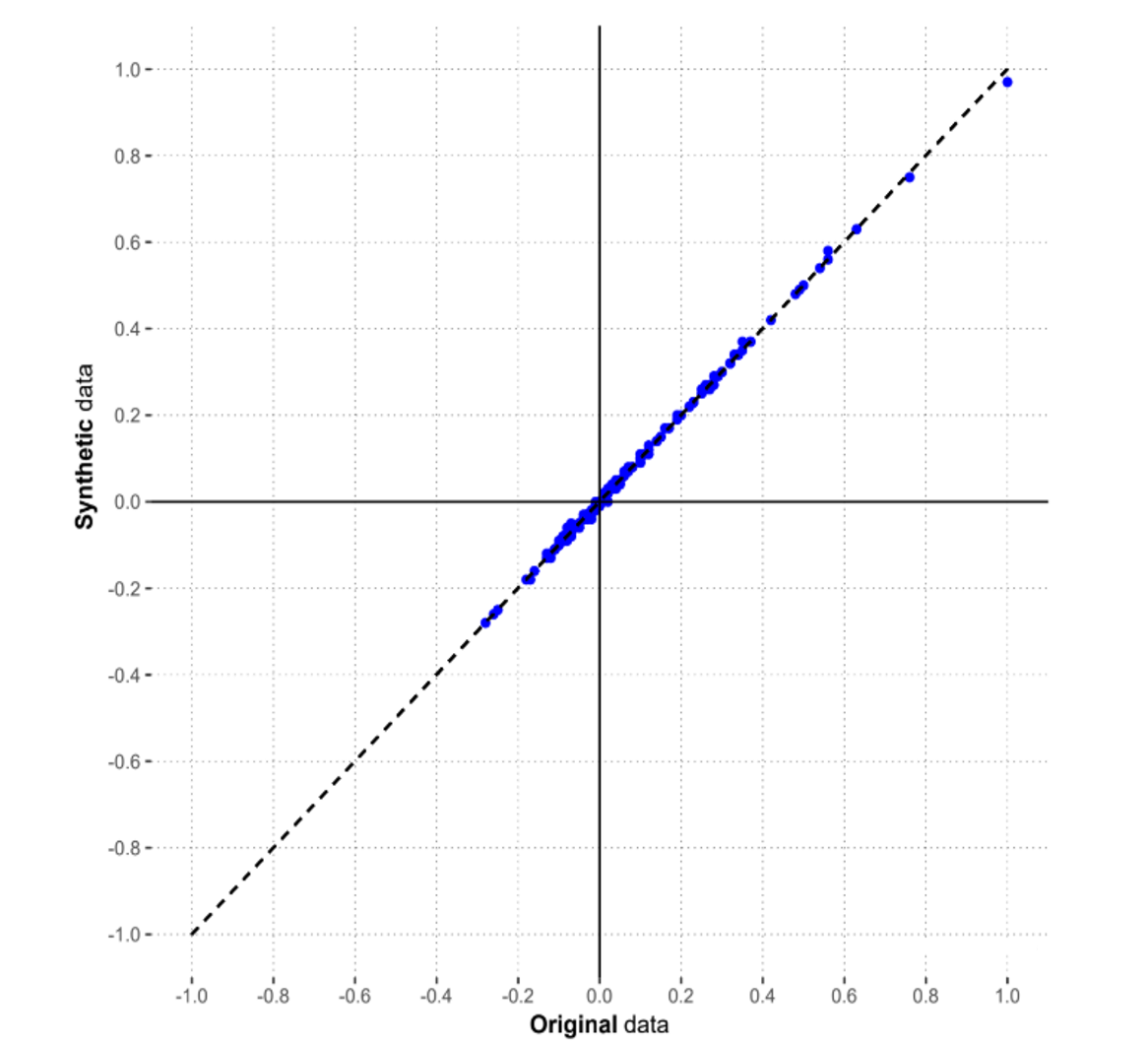


**Figure 1-S:** Spearman correlation coefficients for all pairs of numeric variables, based on the synthetic data (vertical axis) and the original data (horizontal axis). The correlation is preserved for the wide range of correlations, from negative to positive coefficients.

The Second original dataset was extracted from the MIMIC intensive care public database [1], and included three variables that were highly correlated, hemoglobin, hematocrit and patient’s age. As can be seen in Figure 2-S, the high order correlation between hematocrit level, hemoglobin level and age, is consistent between the original data and the synthetic data. The delicate decline of hemoglobin level as patients become older, subject to the increase of hemoglobin level with hematocrit level, in general and within age group, is well preserved by the synthetic data, indicating that association from first order as well as higher order are well preserved by the synthetic data.


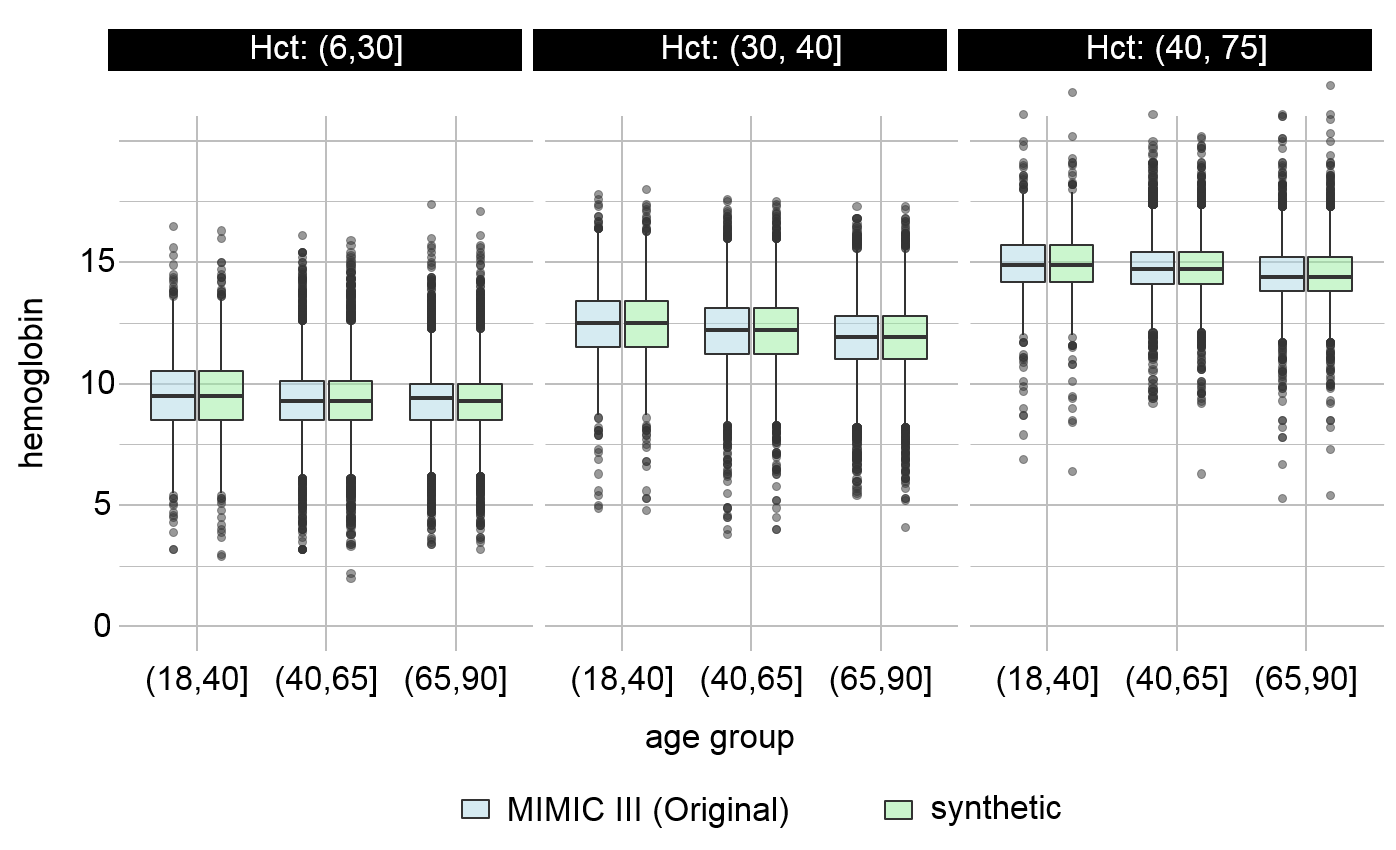


**Figure 2-S:** Boxplot of hemoglobin levels - comparison of MIMIC III (Original) and the synthetic datasets, by patient's age and hematocrit level. The high order correlation is consistent between the original data and the synthetic data. The delicate decline of hemoglobin as age increases is well preserved by the synthetic data within all subgroups.

The above synthetic data generator may be compared to the OSIM health care data simulator [2], which offers to synthesize data related to disease and drug, based on probability distributions estimated from the real data, while accounting for time, gender and age. The relationships between disease and drug are characterized by a single-state transition probabilities that are estimated from the data. In contrast to the limited scope of features that OSIM can address, the MDClone system is suitable for synthetization of any type of data, maximizing the scope of questions that can be studied within the hospital environment, such as the role of lab results as disease risk factors or diagnosis indicators, and readmission and survival outcomes of procedures and hospitalization.

Relationships are restricted by OSIM to behave in a specific format as reflected by the estimated transitional probability matrix, thereby limiting the ability to reflect other and more complex relationships. Furthermore, some degree of data modification and user interference are enabled by the OSIM system. For instance, the user can provide an additional transition table for drug treatment effects. In addition, continuous variables such as age condition count are typically entered as stratified variables. In contrast, the MDClone system seamlessly synthesizes the data based on the source real-world dataset, maintaining high-order relationships for all features included, without lowering the resolution of the source data or making user-driven distributional assumptions.

## References

1. Johnson, AEW, Pollard, TJ, Shen, L, Lehman, L, Feng, M, Ghassemi, M, Moody, B, Szolovits, P, Celi, LA, Mark, RG. MIMIC-III, a freely accessible critical care database. Scientific Data, 2016; doi:10.1038/sdata.2016.35.
2. Murray, RE, Ryan, PB, Reisinger, SJ. Design and validation of a data simulation model for longitudinal healthcare data. AMIA Annual Symposium Proceedings 2011; 1176–1185. PMID: 22195178.
