# Supplementary figures and images for: Analyzing Medical Research Results Based on Synthetic Data and Their Relation to Real Data Results: Systematic Comparison From Five Observational Studies
Source: JMIR Med Inform. 2020 Feb 20;8(2):e16492. doi: 10.2196/16492 (PMC7059086; doi:10.2196/16492)

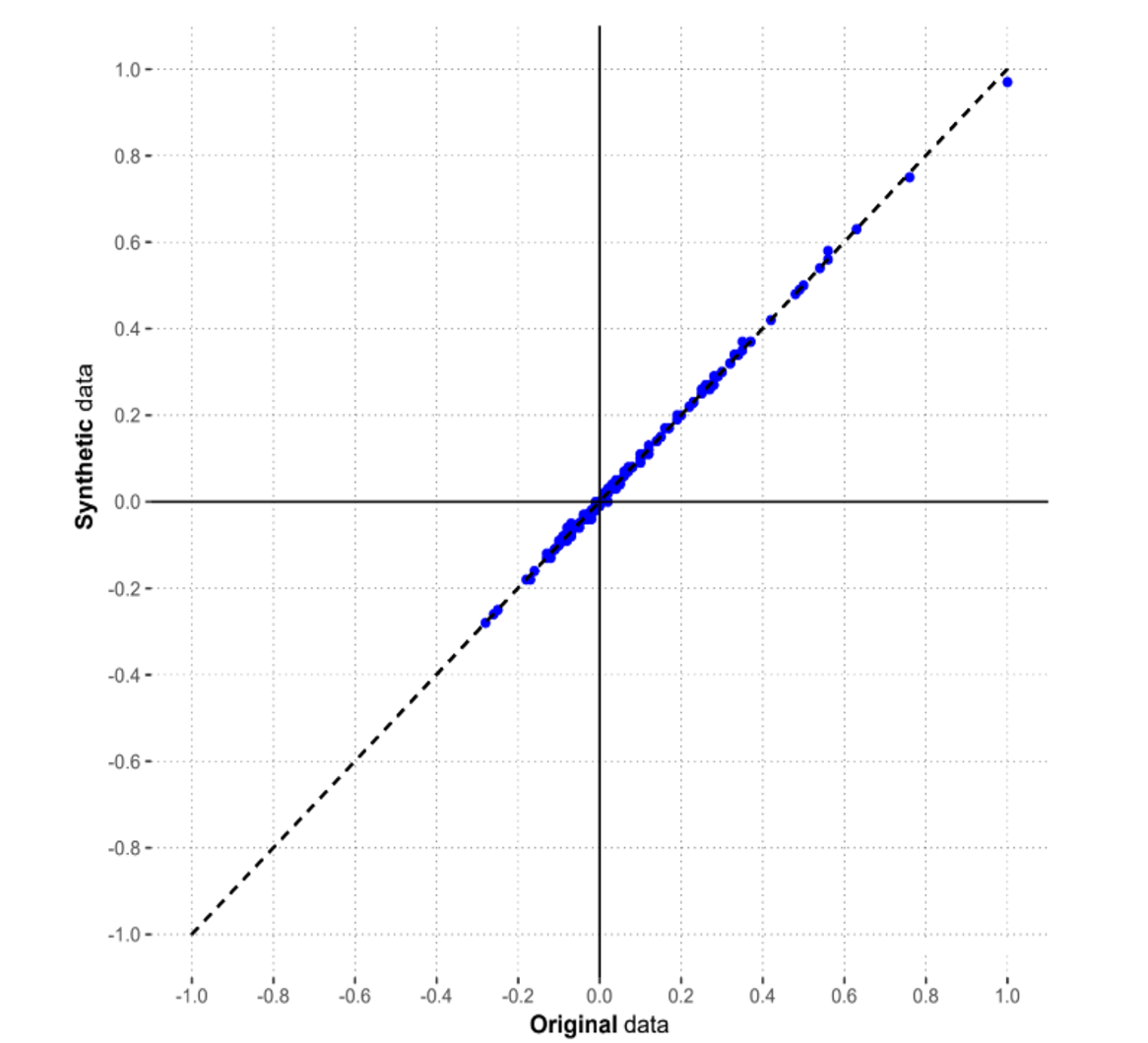

Supplement: Multimedia Appendix 2 [file medinform_v8i2e16492_app2.png]

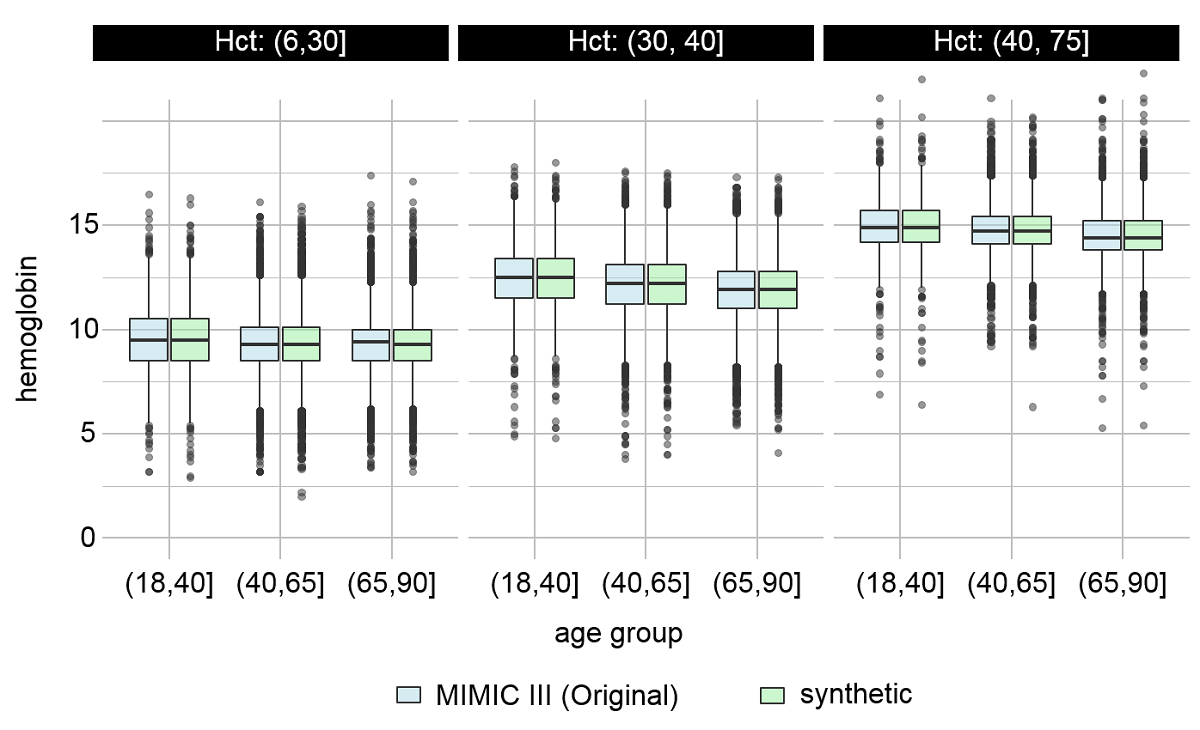

Supplement: Multimedia Appendix 3 [file medinform_v8i2e16492_app3.png]
