# Supplementary material for: Analyzing Medical Research Results Based on Synthetic Data and Their Relation to Real Data Results: Systematic Comparison From Five Observational Studies
Source: JMIR Med Inform. 2020 Feb 20;8(2):e16492. doi: 10.2196/16492 (PMC7059086; doi:10.2196/16492)
Supplement: Multimedia Appendix 4 [file medinform_v8i2e16492_app4.docx]

Table 1-S. Data Characteristics – PPI Prescription Study

| Antiplatelet treatment | All wards | | | Internal Ward | | | Surgery Ward | | |
| --- | --- | --- | --- | --- | --- | --- | --- | --- | --- |
|  | All AOC recipients (n=12,188) | age>65 (n=8,313) | received steroids (n=1,095) | All AOC recipients (n=5,771) | age>65 (n=4,274) | received steroids (n=588) | All AOC recipients (n=1,205) | age>65 (n=693) | received steroids (n=111) |
| AT1 | 3,953 (32.4%) | 2914 (35.1%) | 380 (34.7%) | 2,176 (37.7%) | 1,730 (40.5%) | 241 (40.1%) | 456 (37.8%) | 280 (40.4%) | 32 (28.8%) |
| AT2 | 882 (7.2%) | 642 (7.7%) | 94 (8.6%) | 458 (7.9%) | 362 (8.5%) | 52 (8.8%) | 46 (3.8%) | 25 (3.6%) | 7 (6.3%) |
| DAT | 417 (3.4%) | 281 (3.4%) | 41 (3.7%) | 196 (3.4%) | 147 (3.4%) | 20 (3.4%) | 24 (2.0%) | 14 (2.0%) | 3 (2.7%) |
