# Supplementary material for: Analyzing Medical Research Results Based on Synthetic Data and Their Relation to Real Data Results: Systematic Comparison From Five Observational Studies
Source: JMIR Med Inform. 2020 Feb 20;8(2):e16492. doi: 10.2196/16492 (PMC7059086; doi:10.2196/16492)
Supplement: Multimedia Appendix 5 [file medinform_v8i2e16492_app5.docx]

Table 2-S. Data Characteristics – PCI-STEMI Study

|  | STEMI patients who underwent primary PCI  (n=597) |
| --- | --- |
| Age, years | 59.7 ± 11.8 |
| Gender, male - n (%) | 486 (81.4%) |
| year - n (%) | 2013 (19.1%)  2014 (27.3%)  2015 (28.0%)  2016 (25.6%) |
| Time to PCI (minutes) | 73.2 ± 53.7 |
| Blood urea nitrogen (BUN) (mg/dL) | 17.2 ± 6.9 |
| Creatinine (mg/dL) | 0.96 ± 0.37 |
| Hemoglobin (g/dL) | 13.7 ± 1.8 |
| Glomerular filtration rate (GFR) (mL/min/1.73m^2^) | 83.1 ± 23.8 |
| Severe cardiac presentation - n (%) | 48 (8.0%) |
| Prior ischemic heart disease (IHD) - n (%) | 60 (10.1%) |
| Treated with Aggrastat at home - n (%) | 172 (28.8%) |
