# Supplementary material for: Analyzing Medical Research Results Based on Synthetic Data and Their Relation to Real Data Results: Systematic Comparison From Five Observational Studies
Source: JMIR Med Inform. 2020 Feb 20;8(2):e16492. doi: 10.2196/16492 (PMC7059086; doi:10.2196/16492)
Supplement: Multimedia Appendix 6 [file medinform_v8i2e16492_app6.docx]

Table 3-S. Data Characteristics – BUN-ADHF Study

|  | Inpatients with primary diagnosis of heart failure  who survived to discharge  (n=4,590) |
| --- | --- |
| Age, years | 74.2 ± 12.1 |
| Gender, male - n (%) | 2,496 (54.4%) |
| Length of stay (days) | 7.2 ± 8.5 |
| Diabetes - n (%) | 2,470 (53.8%) |
| Hypertension - n (%) | 3,811 (83.0%) |
| Chronic obstructive pulmonary disease (COPD) - n (%) | 684 (14.9%) |
| Peripheral vascular disease (PVD) - n (%) | 545 (11.9%) |
| Chronic kidney disease (CKD) - n (%) | 1,176 (25.6%) |
| Ischemic heart disease (IHD) - n (%) | 2,851 (62.1%) |
| Atrial fibrillation (AF) - n (%) | 1,912 (41.7%) |
| Valvular heart disease (VHD) - n (%) | 1,309 (28.5%) |
| Pulmonary hypertension (PHTN) - n (%) | 1,046 (22.8%) |
| Blood urea nitrogen (BUN) on admission (mg/dL) | 30.9 ± 17.9 |
| Blood urea nitrogen (BUN) on discharge (mg/dL) | 34.1 ± 18.8 |
| Creatinine on admission (mg/dL) | 1.53 ± 1.04 |
| Creatinine on discharge (mg/dL) | 1.5 ± 1.0 |
| Glomerular filtration rate (GFR) (mL/min/1.73m^2^) | 51.6 ± 24.3 |
| Red blood cell distribution width (RDW) | 15.7 ± 2.0 |
| White blood cells (WBC) | 10.1 ± 6.3 |
| Platelets | 240 ± 98 |
| Bilirubin (mg/dL) | 0.7 ± 0.6 |
| Sodium (mEq/L) | 137 ± 4 |
| Potassium (mEq/L) | 4.3 ± 0.6 |
