# Supplementary material for: Analyzing Medical Research Results Based on Synthetic Data and Their Relation to Real Data Results: Systematic Comparison From Five Observational Studies
Source: JMIR Med Inform. 2020 Feb 20;8(2):e16492. doi: 10.2196/16492 (PMC7059086; doi:10.2196/16492)
Supplement: Multimedia Appendix 7 [file medinform_v8i2e16492_app7.docx]

Table 4-S. Data Characteristics – Hypoglycemia Insulin Study

|  | Glargine | Detemir | p-value |
| --- | --- | --- | --- |
|  | (n=3,843) | (n=834) |  |
| Age, years | 67.2 ± 13.9 | 65.3 ± 13.6 | <0.0001 |
| Gender, male | 2,118  (56.9%) | 427  (51.2%) | 0.043 |
| Weight ( kg) | 84.3 ± 20.4 | 86.1 ± 20.4 | 0.031 |
| Medical wards (% Internal) | 2,848  (74.1%) | 648  (77.7%) | 0.0341 |
| Length of stay (days) | 12.9 ± 17.2 | 11.4 ± 14.9 | 0.022 |
| Maximal creatinine (mg/dL) | 1.6 ± 1.4 | 1.6 ± 1.4 | 0.839 |
| Minimal albumin (g/dL) | 2.6 ± 0.74 | 2.8 ± 0.7 | <0.0001 |
| Minimal glucose (mg/dL) | 111.9 ± 46.9 | 104.9 ± 45.6 | <0.0001 |
| Treated with insulin at home - n (%) | 1,970  (51.3%) | 555  (66.5%) | <0.0001 |
| Basal insulin dose (units) | 20.8 ± 11.7 | 23.1 ± 13.3 | <0.0001 |
| Insulin to weight ratio (unit/kg) | 0.33 ± 4.3 | 0.27 ± 0.15 | 0.723 |
| Use of rapid insulin - n (%) | 3,243  (84.4%) | 670  (80.3%) | 0.005 |
